# Supplementary figures and images for: Effects of self-administered binaural beats on meditative and introspective states
Source: PLoS One. 2026 Apr 1;21(4):e0335580. doi: 10.1371/journal.pone.0335580 (PMC13042839; doi:10.1371/journal.pone.0335580)

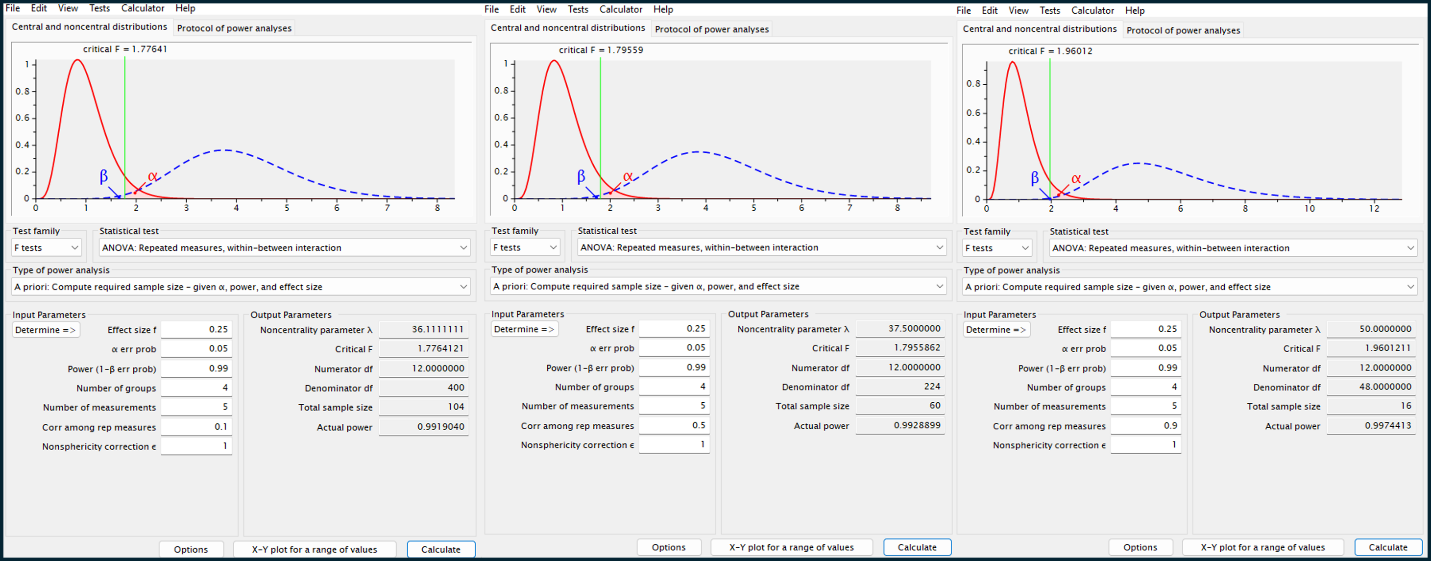

Supplement: S1 Fig — Power analyses for a two-way (4 x 5) ANOVA on GPower with autocorrelation coefficients set to. 1, 5 or 9. (TIF) [file pone.0335580.s001.tif]

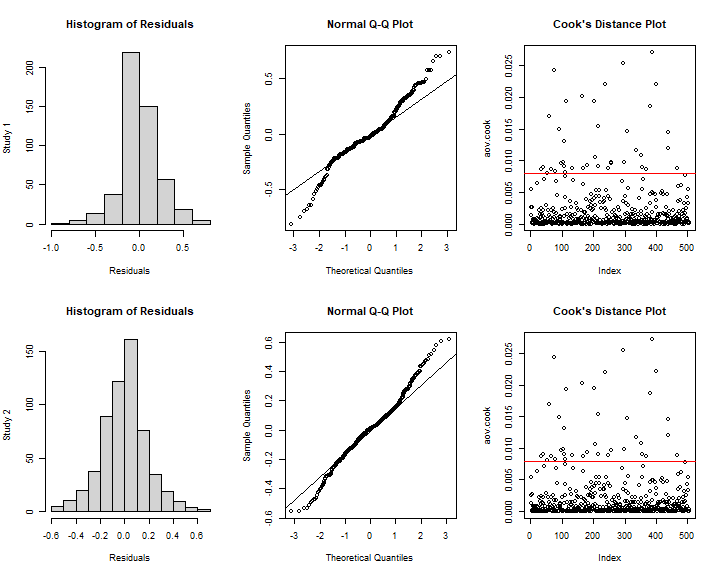

Supplement: S4 Fig — Histogram and QQ plots of residuals from the linear mixed-effects model for Study 1 (top row) and Study 2 (bottom row). A Cook's distance threshold of 4/n (rightmost column, red intercept) identified significant outliers in the initial model, which were excluded from the outlier-removed datasets. (TIF) [file pone.0335580.s004.tif]

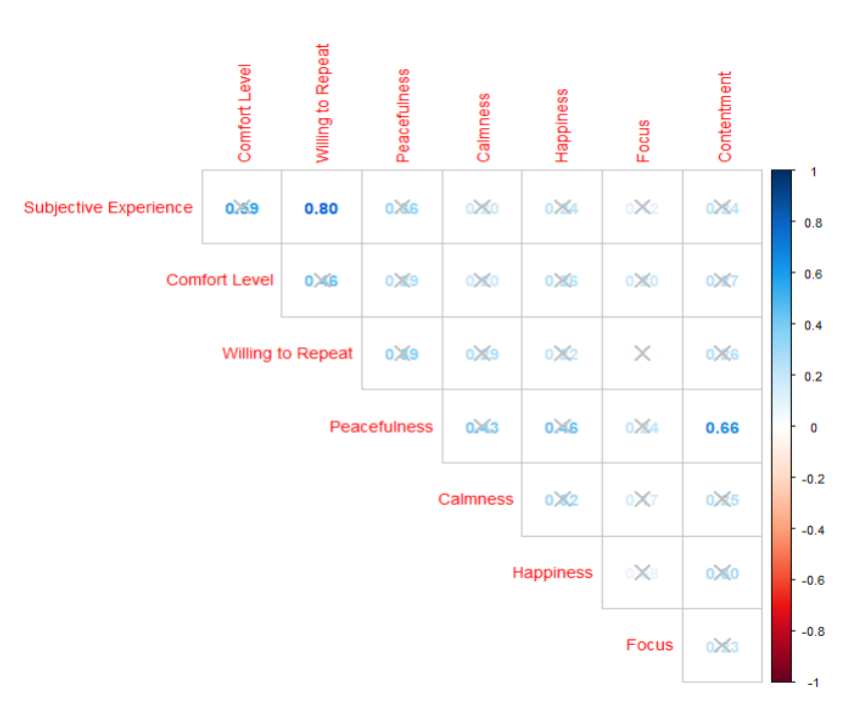

Supplement: S8 Fig — Correlation matrix across mood-state change scores (peacefulness, calmness, happiness, focus, contentment) and sentiment coded responses to questions about introspective experiences (comfort level, willingness to repeat, subjective experience). (TIFF) [file pone.0335580.s008.tiff]

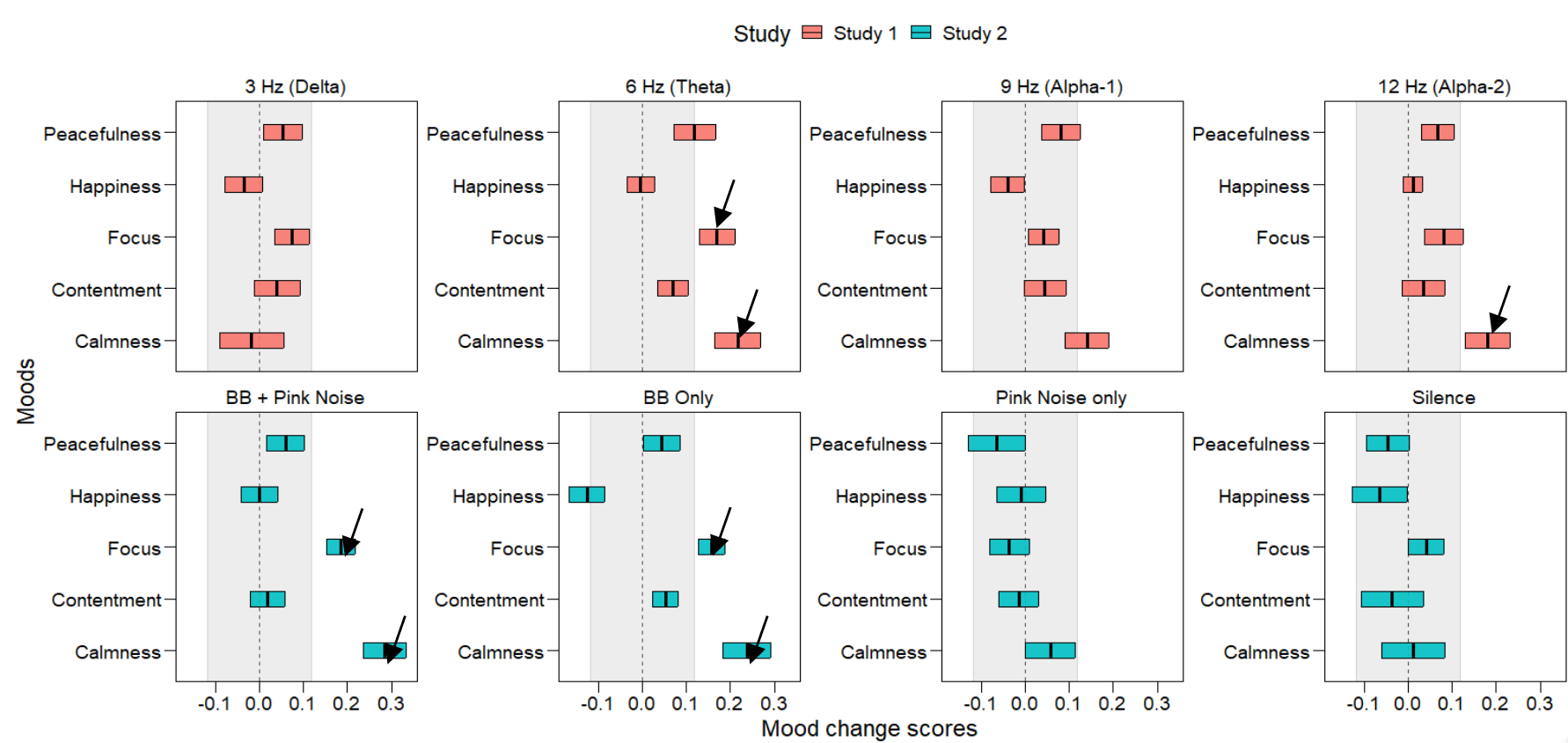

Supplement: S10 Fig — Crossbar plots indicating mean changes with 95% confidence intervals (CIs) across moods (y-axis) for the full datasets across Study 1 (row 1) and Study 2 (row 2). Bars with CIs that do not overlap ROPEs (±.127) indicate practically meaningful mood changes (marked). (TIFF) [file pone.0335580.s010.tiff]
