# Supplementary material for: Effects of self-administered binaural beats on meditative and introspective states
Source: PLoS One. 2026 Apr 1;21(4):e0335580. doi: 10.1371/journal.pone.0335580 (PMC13042839; doi:10.1371/journal.pone.0335580)
Supplement: S2 File — Prerecorded speech segments presented during different phases of the study. (DOCX) [file pone.0335580.s002.docx]

**S2: Experimenter transcript**

*Prerecorded speech segments presented during different phases of the study.*

--{*Transcript for both studies*}--

Hi. Thank you for choosing to take part in this study! In this study, you will undergo a form of auditory entrainment involving binaural tones and report its impact on your current mood. You will hear two tones of different frequencies presented to each ear for five minutes, and report your mood before and after hearing the tones. The entire study should take less than fifteen minutes to complete. First, please navigate to the link provided on the screen.

{*Wait for participant to access the application*}

Please scroll to the bottom of the page until you see the Auditory Entrainment Module. This is the main interface you will interact with. Please now put on the head phones provided.

{*Wait for participant to place headphones: the researcher then plays a test tone*}

Please set the volume to a comfortable range. You should be able to hear the test tone clearly but not too loud. Once you have set a comfortable volume, let me know.

{*Wait for participant to indicate comfort level*}

Now, let us set up the parameters. Please set the parameters for Duration to five minutes. Then, please set the ‘Carrier Frequency’ to two hundred and fifty Hertz by moving the slider.

{*Remaining instructions depended on participant group allocation – detailed in* **S3**}

Now that the parameters have been set up, please continue with the application and answer honestly the questions provided about your current mood.

{*Participants complete the first mood-state VAS*}

Please ensure your headphones are placed comfortably, then press the Start button. Please remember to adjust the volume so the tone is clear but not uncomfortably loud. Now you can press the ‘Start Auditory Session’ to begin.

{*Participants commenced the auditory phase for five minutes, indicated by an on-screen timer*}

Thank you for listening. Please now indicate your current mood once again, and complete any additional items that appear.

{*Participants complete the second mood-state VAS + additional items, detailed in* **S3**}

Thanks for taking part! Please click on the Final Submit button if you haven’t already.

{*End of participation*}
