# Supplementary material for: Effects of self-administered binaural beats on meditative and introspective states
Source: PLoS One. 2026 Apr 1;21(4):e0335580. doi: 10.1371/journal.pone.0335580 (PMC13042839; doi:10.1371/journal.pone.0335580)
Supplement: S3 File — Surveys deployed across studies. (DOCX) [file pone.0335580.s003.docx]

**S3: Survey Items**

*Surveys deployed across studies*

**Mood Visual Analog Scales (VAS)**:

{*Each VAS item comprised 9-point slider scales with labels on the left, center and right*}

- How HAPPY or UNHAPPY do YOU feel RIGHT NOW? {1 - Very Unhappy, 2, 3, 4, 5 - Neither Happy nor Unhappy, 6, 7, 8 ,9 - Very Happy}
- How CALM or STRESSED do YOU feel RIGHT NOW? {1 - Very Calm, 2, 3, 4, 5 - Neither Calm nor Stressed, 6, 7, 8 ,9 - Very Stressed}
- How AGITATED or PEACEFUL do YOU feel RIGHT NOW? {1 - Very Agitated, 2, 3, 4, 5 - Neither Agitated nor Peaceful, 6, 7, 8, 9 - Very Peaceful}
- How FOCUSED or DISTRACTED do YOU feel RIGHT NOW? {1 - Very Distracted, 2, 3, 4, 5 - Neither Focused nor Distracted, 6, 7, 8, 9 - Very Focused}
- How CONTENT or FRUSTRATED do you feel RIGHT NOW? {1 - Very Frustrated, 2, 3, 4, 5 - Neither Content nor Frustrated, 6, 7, 8, 9 - Very Content}

**Demographic and Laterality Surveys**:

- Please type in how old you are: (numeric values only)
- Please indicate your sex: (options: Male, Female, Other)
- When you hold a phone to your head for a call, which ear do you usually listen with? (Left, Right, Either Ear Equally)

**Group Assignment Instructions** (from transcript):

{*For Study 1*} Please adjust the Binaural Beat Frequency to **SIX** (**THREE, NINE** or **TWELVE)** Hertz and press start

{*For Study 2*) Please adjust the Tone Type to **Binaural Beats** (or **Pink Noise**). After setting the **binaural tone to 6 Hz** (or the **pink noise volume to 30%**), please press start.
