# Supplementary material for: Effects of self-administered binaural beats on meditative and introspective states
Source: PLoS One. 2026 Apr 1;21(4):e0335580. doi: 10.1371/journal.pone.0335580 (PMC13042839; doi:10.1371/journal.pone.0335580)
Supplement: S5 Table — (DOCX) [file pone.0335580.s005.docx]

**S5: Post-hoc test outcomes across Study 1**

| Significant *fdr*-corrected Tukey HSD tests on the full and outlier-removed datasets across Study 1 | | | | | | | |
| --- | --- | --- | --- | --- | --- | --- | --- |
| **Dataset** | **Term** | **Group 1** | **Group 2** | **Estimate** | **95% Low** | **95% High** | ***p* (adj)** |
| Full | Group | 3 Hz (Delta) | 6 Hz (Theta) | 0.09 | 0.02 | 0.16 | 0.01 |
| Full | Mood | Calmness | Happiness | -0.15 | -0.23 | -0.06 | 0 |
| Full | Mood | Focus | Happiness | -0.11 | -0.19 | -0.02 | 0 |
| Full | Mood | Happiness | Peacefulness | 0.1 | 0.01 | 0.18 | 0.01 |
| Full | Group:Mood | 3 Hz (Delta):Calmness | 6 Hz (Theta):Calmness | 0.23 | 0.01 | 0.46 | 0.03 |
| Full | Group:Mood | 6 Hz (Theta):Calmness | 3 Hz (Delta):Happiness | -0.25 | -0.48 | -0.03 | 0.01 |
| Full | Group:Mood | 6 Hz (Theta):Calmness | 9 Hz (Alpha-1):Happiness | -0.26 | -0.48 | -0.04 | 0.01 |
| Full | Group:Mood | 12 Hz (Alpha-2):Calmness | 9 Hz (Alpha-1):Happiness | -0.22 | -0.44 | 0 | 0.04 |
| No-Outliers | Group | 3 Hz (Delta) | 6 Hz (Theta) | 0.09 | 0.03 | 0.14 | 0 |
| No-Outliers | Group | 6 Hz (Theta) | 9 Hz (Alpha-1) | -0.06 | -0.11 | 0 | 0.04 |
| No-Outliers | Mood | Calmness | Contentment | -0.08 | -0.15 | -0.02 | 0.01 |
| No-Outliers | Mood | Calmness | Happiness | -0.14 | -0.2 | -0.07 | 0 |
| No-Outliers | Mood | Calmness | Peacefulness | -0.07 | -0.14 | -0.01 | 0.02 |
| No-Outliers | Mood | Focus | Happiness | -0.08 | -0.15 | -0.02 | 0 |
| No-Outliers | Mood | Happiness | Peacefulness | 0.07 | 0 | 0.13 | 0.03 |
| No-Outliers | Group:Mood | 3 Hz (Delta):Calmness | 6 Hz (Theta):Focus | 0.19 | 0.01 | 0.37 | 0.03 |
| No-Outliers | Group:Mood | 6 Hz (Theta):Calmness | 3 Hz (Delta):Happiness | -0.23 | -0.4 | -0.06 | 0 |
| No-Outliers | Group:Mood | 6 Hz (Theta):Calmness | 9 Hz (Alpha-1):Happiness | -0.18 | -0.35 | -0.01 | 0.02 |
| No-Outliers | Group:Mood | 9 Hz (Alpha-1):Calmness | 3 Hz (Delta):Happiness | -0.21 | -0.38 | -0.04 | 0 |
| No-Outliers | Group:Mood | 12 Hz (Alpha-2):Calmness | 3 Hz (Delta):Happiness | -0.23 | -0.4 | -0.06 | 0 |
| No-Outliers | Group:Mood | 12 Hz (Alpha-2):Calmness | 9 Hz (Alpha-1):Happiness | -0.18 | -0.35 | -0.02 | 0.01 |
| No-Outliers | Group:Mood | 12 Hz (Alpha-2):Calmness | 12 Hz (Alpha-2):Happiness | -0.17 | -0.33 | 0 | 0.04 |
| No-Outliers | Group:Mood | 9 Hz (Alpha-1):Contentment | 6 Hz (Theta):Focus | 0.18 | 0.01 | 0.34 | 0.03 |
| No-Outliers | Group:Mood | 6 Hz (Theta):Focus | 9 Hz (Alpha-1):Focus | -0.16 | -0.33 | 0 | 0.04 |
| No-Outliers | Group:Mood | 6 Hz (Theta):Focus | 3 Hz (Delta):Happiness | -0.24 | -0.41 | -0.07 | 0 |
| No-Outliers | Group:Mood | 6 Hz (Theta):Focus | 6 Hz (Theta):Happiness | -0.17 | -0.34 | 0 | 0.04 |
| No-Outliers | Group:Mood | 6 Hz (Theta):Focus | 9 Hz (Alpha-1):Happiness | -0.19 | -0.36 | -0.03 | 0.01 |
| No-Outliers | Group:Mood | 6 Hz (Theta):Focus | 12 Hz (Alpha-2):Happiness | -0.18 | -0.34 | -0.01 | 0.02 |
| No-Outliers | Group:Mood | 6 Hz (Theta):Focus | 3 Hz (Delta):Peacefulness | -0.17 | -0.34 | -0.01 | 0.03 |
