# Supplementary material for: Effects of self-administered binaural beats on meditative and introspective states
Source: PLoS One. 2026 Apr 1;21(4):e0335580. doi: 10.1371/journal.pone.0335580 (PMC13042839; doi:10.1371/journal.pone.0335580)
Supplement: S6 Table — Significant fdr-corrected Tukey HSD tests on the full and outlier-removed datasets across Study 2. (DOCX) [file pone.0335580.s006.docx]

**S5: Post-hoc test outcomes across Study 2**

| Significant *fdr*-corrected Tukey HSD tests on the full and outlier-removed datasets across Study 2 | | | | | | | |
| --- | --- | --- | --- | --- | --- | --- | --- |
| **Dataset** | **Term** | **Group 1** | **Group 2** | **Estimate** | **95% Low** | **95% High** | ***p* (adj)** |
| Full | Group | BB + Pink Noise | Pink Noise only | -0.12 | -0.2 | -0.05 | 0 |
| Full | Group | BB + Pink Noise | Silence | -0.13 | -0.21 | -0.05 | 0 |
| Full | Group | BB Only | Pink Noise only | -0.09 | -0.16 | -0.01 | 0.01 |
| Full | Group | BB Only | Silence | -0.09 | -0.17 | -0.01 | 0.02 |
| Full | Mood | Calmness | Contentment | -0.16 | -0.25 | -0.07 | 0 |
| Full | Mood | Calmness | Happiness | -0.22 | -0.31 | -0.13 | 0 |
| Full | Mood | Calmness | Peacefulness | -0.16 | -0.25 | -0.07 | 0 |
| Full | Mood | Focus | Happiness | -0.15 | -0.24 | -0.06 | 0 |
| Full | Mood | Focus | Peacefulness | -0.09 | -0.18 | 0 | 0.04 |
| Full | Group:Mood | BB + Pink Noise:Calmness | Silence:Calmness | -0.27 | -0.52 | -0.03 | 0.01 |
| Full | Group:Mood | BB + Pink Noise:Calmness | BB + Pink Noise:Contentment | -0.27 | -0.48 | -0.05 | 0 |
| Full | Group:Mood | BB + Pink Noise:Calmness | BB Only:Contentment | -0.23 | -0.45 | -0.02 | 0.02 |
| Full | Group:Mood | BB + Pink Noise:Calmness | Pink Noise only:Contentment | -0.3 | -0.53 | -0.07 | 0 |
| Full | Group:Mood | BB + Pink Noise:Calmness | Silence:Contentment | -0.32 | -0.57 | -0.08 | 0 |
| Full | Group:Mood | BB + Pink Noise:Calmness | Pink Noise only:Focus | -0.32 | -0.55 | -0.09 | 0 |
| Full | Group:Mood | BB + Pink Noise:Calmness | BB + Pink Noise:Happiness | -0.29 | -0.5 | -0.07 | 0 |
| Full | Group:Mood | BB + Pink Noise:Calmness | BB Only:Happiness | -0.41 | -0.63 | -0.19 | 0 |
| Full | Group:Mood | BB + Pink Noise:Calmness | Pink Noise only:Happiness | -0.29 | -0.52 | -0.06 | 0 |
| Full | Group:Mood | BB + Pink Noise:Calmness | Silence:Happiness | -0.35 | -0.6 | -0.1 | 0 |
| Full | Group:Mood | BB + Pink Noise:Calmness | BB + Pink Noise:Peacefulness | -0.23 | -0.44 | -0.01 | 0.03 |
| Full | Group:Mood | BB + Pink Noise:Calmness | BB Only:Peacefulness | -0.24 | -0.46 | -0.02 | 0.01 |
| Full | Group:Mood | BB + Pink Noise:Calmness | Pink Noise only:Peacefulness | -0.35 | -0.58 | -0.12 | 0 |
| Full | Group:Mood | BB + Pink Noise:Calmness | Silence:Peacefulness | -0.33 | -0.58 | -0.09 | 0 |
| Full | Group:Mood | BB Only:Calmness | BB + Pink Noise:Contentment | -0.22 | -0.44 | 0 | 0.04 |
| Full | Group:Mood | BB Only:Calmness | Pink Noise only:Contentment | -0.25 | -0.48 | -0.02 | 0.01 |
| Full | Group:Mood | BB Only:Calmness | Silence:Contentment | -0.27 | -0.52 | -0.03 | 0.01 |
| Full | Group:Mood | BB Only:Calmness | Pink Noise only:Focus | -0.28 | -0.5 | -0.05 | 0 |
| Full | Group:Mood | BB Only:Calmness | BB + Pink Noise:Happiness | -0.24 | -0.46 | -0.02 | 0.02 |
| Full | Group:Mood | BB Only:Calmness | BB Only:Happiness | -0.36 | -0.58 | -0.15 | 0 |
| Full | Group:Mood | BB Only:Calmness | Pink Noise only:Happiness | -0.25 | -0.48 | -0.02 | 0.02 |
| Full | Group:Mood | BB Only:Calmness | Silence:Happiness | -0.3 | -0.55 | -0.06 | 0 |
| Full | Group:Mood | BB Only:Calmness | Pink Noise only:Peacefulness | -0.3 | -0.53 | -0.07 | 0 |
| Full | Group:Mood | BB Only:Calmness | Silence:Peacefulness | -0.28 | -0.53 | -0.04 | 0.01 |
| Full | Group:Mood | BB + Pink Noise:Focus | BB Only:Happiness | -0.31 | -0.53 | -0.09 | 0 |
| Full | Group:Mood | BB + Pink Noise:Focus | Silence:Happiness | -0.25 | -0.5 | 0 | 0.04 |
| Full | Group:Mood | BB + Pink Noise:Focus | Pink Noise only:Peacefulness | -0.25 | -0.48 | -0.02 | 0.02 |
| Full | Group:Mood | BB Only:Focus | BB Only:Happiness | -0.28 | -0.5 | -0.07 | 0 |
| No-Outliers | Group | BB + Pink Noise | Pink Noise only | -0.09 | -0.14 | -0.05 | 0 |
| No-Outliers | Group | BB + Pink Noise | Silence | -0.09 | -0.14 | -0.04 | 0 |
| No-Outliers | Group | BB Only | Pink Noise only | -0.07 | -0.12 | -0.02 | 0 |
| No-Outliers | Group | BB Only | Silence | -0.07 | -0.12 | -0.01 | 0.01 |
| No-Outliers | Mood | Calmness | Contentment | -0.12 | -0.19 | -0.06 | 0 |
| No-Outliers | Mood | Calmness | Happiness | -0.15 | -0.21 | -0.09 | 0 |
| No-Outliers | Mood | Calmness | Peacefulness | -0.13 | -0.19 | -0.07 | 0 |
| No-Outliers | Mood | Contentment | Focus | 0.08 | 0.02 | 0.13 | 0 |
| No-Outliers | Mood | Focus | Happiness | -0.1 | -0.16 | -0.05 | 0 |
| No-Outliers | Mood | Focus | Peacefulness | -0.08 | -0.14 | -0.02 | 0 |
| No-Outliers | Group:Mood | BB + Pink Noise:Calmness | Silence:Calmness | -0.21 | -0.4 | -0.02 | 0.01 |
| No-Outliers | Group:Mood | BB + Pink Noise:Calmness | BB + Pink Noise:Contentment | -0.2 | -0.35 | -0.04 | 0 |
| No-Outliers | Group:Mood | BB + Pink Noise:Calmness | BB Only:Contentment | -0.18 | -0.33 | -0.02 | 0.01 |
| No-Outliers | Group:Mood | BB + Pink Noise:Calmness | Pink Noise only:Contentment | -0.24 | -0.41 | -0.08 | 0 |
| No-Outliers | Group:Mood | BB + Pink Noise:Calmness | Silence:Contentment | -0.23 | -0.41 | -0.05 | 0 |
| No-Outliers | Group:Mood | BB + Pink Noise:Calmness | Pink Noise only:Focus | -0.24 | -0.41 | -0.08 | 0 |
| No-Outliers | Group:Mood | BB + Pink Noise:Calmness | Silence:Focus | -0.22 | -0.39 | -0.05 | 0 |
| No-Outliers | Group:Mood | BB + Pink Noise:Calmness | BB + Pink Noise:Happiness | -0.19 | -0.35 | -0.04 | 0 |
| No-Outliers | Group:Mood | BB + Pink Noise:Calmness | BB Only:Happiness | -0.33 | -0.51 | -0.16 | 0 |
| No-Outliers | Group:Mood | BB + Pink Noise:Calmness | Pink Noise only:Happiness | -0.24 | -0.41 | -0.07 | 0 |
| No-Outliers | Group:Mood | BB + Pink Noise:Calmness | Silence:Happiness | -0.21 | -0.39 | -0.04 | 0 |
| No-Outliers | Group:Mood | BB + Pink Noise:Calmness | BB + Pink Noise:Peacefulness | -0.18 | -0.33 | -0.02 | 0.01 |
| No-Outliers | Group:Mood | BB + Pink Noise:Calmness | BB Only:Peacefulness | -0.17 | -0.33 | -0.02 | 0.01 |
| No-Outliers | Group:Mood | BB + Pink Noise:Calmness | Pink Noise only:Peacefulness | -0.3 | -0.48 | -0.12 | 0 |
| No-Outliers | Group:Mood | BB + Pink Noise:Calmness | Silence:Peacefulness | -0.24 | -0.41 | -0.08 | 0 |
| No-Outliers | Group:Mood | BB Only:Calmness | BB + Pink Noise:Contentment | -0.17 | -0.34 | -0.01 | 0.02 |
| No-Outliers | Group:Mood | BB Only:Calmness | Pink Noise only:Contentment | -0.22 | -0.39 | -0.05 | 0 |
| No-Outliers | Group:Mood | BB Only:Calmness | Silence:Contentment | -0.21 | -0.39 | -0.03 | 0.01 |
| No-Outliers | Group:Mood | BB Only:Calmness | Pink Noise only:Focus | -0.22 | -0.39 | -0.05 | 0 |
| No-Outliers | Group:Mood | BB Only:Calmness | Silence:Focus | -0.19 | -0.37 | -0.02 | 0.01 |
| No-Outliers | Group:Mood | BB Only:Calmness | BB + Pink Noise:Happiness | -0.17 | -0.33 | -0.01 | 0.02 |
| No-Outliers | Group:Mood | BB Only:Calmness | BB Only:Happiness | -0.31 | -0.49 | -0.14 | 0 |
| No-Outliers | Group:Mood | BB Only:Calmness | Pink Noise only:Happiness | -0.22 | -0.39 | -0.05 | 0 |
| No-Outliers | Group:Mood | BB Only:Calmness | Silence:Happiness | -0.19 | -0.37 | -0.01 | 0.02 |
| No-Outliers | Group:Mood | BB Only:Calmness | Pink Noise only:Peacefulness | -0.28 | -0.46 | -0.1 | 0 |
| No-Outliers | Group:Mood | BB Only:Calmness | Silence:Peacefulness | -0.22 | -0.39 | -0.05 | 0 |
| No-Outliers | Group:Mood | Pink Noise only:Calmness | BB Only:Happiness | -0.19 | -0.36 | -0.03 | 0 |
| No-Outliers | Group:Mood | Silence:Calmness | BB + Pink Noise:Focus | 0.2 | 0.02 | 0.37 | 0.01 |
| No-Outliers | Group:Mood | BB + Pink Noise:Contentment | BB + Pink Noise:Focus | 0.18 | 0.04 | 0.33 | 0 |
| No-Outliers | Group:Mood | BB Only:Contentment | BB + Pink Noise:Focus | 0.16 | 0.01 | 0.31 | 0.02 |
| No-Outliers | Group:Mood | BB Only:Contentment | BB Only:Happiness | -0.16 | -0.31 | -0.01 | 0.03 |
| No-Outliers | Group:Mood | Pink Noise only:Contentment | BB + Pink Noise:Focus | 0.23 | 0.08 | 0.38 | 0 |
| No-Outliers | Group:Mood | Pink Noise only:Contentment | BB Only:Focus | 0.15 | 0.02 | 0.29 | 0.01 |
| No-Outliers | Group:Mood | Silence:Contentment | BB + Pink Noise:Focus | 0.22 | 0.05 | 0.39 | 0 |
| No-Outliers | Group:Mood | BB + Pink Noise:Focus | Pink Noise only:Focus | -0.23 | -0.38 | -0.07 | 0 |
| No-Outliers | Group:Mood | BB + Pink Noise:Focus | Silence:Focus | -0.2 | -0.36 | -0.04 | 0 |
| No-Outliers | Group:Mood | BB + Pink Noise:Focus | BB + Pink Noise:Happiness | -0.18 | -0.32 | -0.03 | 0 |
| No-Outliers | Group:Mood | BB + Pink Noise:Focus | BB Only:Happiness | -0.32 | -0.48 | -0.16 | 0 |
| No-Outliers | Group:Mood | BB + Pink Noise:Focus | Pink Noise only:Happiness | -0.22 | -0.38 | -0.07 | 0 |
| No-Outliers | Group:Mood | BB + Pink Noise:Focus | Silence:Happiness | -0.2 | -0.36 | -0.04 | 0 |
| No-Outliers | Group:Mood | BB + Pink Noise:Focus | BB + Pink Noise:Peacefulness | -0.16 | -0.31 | -0.02 | 0.01 |
| No-Outliers | Group:Mood | BB + Pink Noise:Focus | BB Only:Peacefulness | -0.16 | -0.3 | -0.01 | 0.02 |
| No-Outliers | Group:Mood | BB + Pink Noise:Focus | Pink Noise only:Peacefulness | -0.29 | -0.46 | -0.12 | 0 |
| No-Outliers | Group:Mood | BB + Pink Noise:Focus | Silence:Peacefulness | -0.23 | -0.38 | -0.07 | 0 |
| No-Outliers | Group:Mood | BB Only:Focus | Pink Noise only:Focus | -0.15 | -0.29 | -0.01 | 0.02 |
| No-Outliers | Group:Mood | BB Only:Focus | BB Only:Happiness | -0.24 | -0.4 | -0.09 | 0 |
| No-Outliers | Group:Mood | BB Only:Focus | Pink Noise only:Happiness | -0.15 | -0.29 | -0.01 | 0.03 |
| No-Outliers | Group:Mood | BB Only:Focus | Pink Noise only:Peacefulness | -0.21 | -0.37 | -0.06 | 0 |
| No-Outliers | Group:Mood | BB Only:Focus | Silence:Peacefulness | -0.15 | -0.3 | -0.01 | 0.02 |
| No-Outliers | Group:Mood | BB Only:Happiness | BB + Pink Noise:Peacefulness | 0.16 | 0.01 | 0.31 | 0.03 |
| No-Outliers | Group:Mood | BB Only:Happiness | BB Only:Peacefulness | 0.16 | 0.01 | 0.31 | 0.02 |
