# Supplementary material for: Effects of self-administered binaural beats on meditative and introspective states
Source: PLoS One. 2026 Apr 1;21(4):e0335580. doi: 10.1371/journal.pone.0335580 (PMC13042839; doi:10.1371/journal.pone.0335580)
Supplement: S9 Table — (DOCX) [file pone.0335580.s009.docx]

**S9. Post-hoc tests comparing Mood Change Scores across Coded Sentiments**

| Post-hoc Tukey tests contrasting distributions of mood change scores across coded sentiment categories | | | | | | |
| --- | --- | --- | --- | --- | --- | --- |
| **Moods** | **Group 1** | **Group 2** | **Mean Estimate** | **95% Lower CI** | **95% Upper CI** | ***p*-value (adj.)** |
| **Calmness** | **Negative** | **Uncertain** | **0.117** | **0.006** | **0.228** | **0.036** |
| **Calmness** | **Negative** | **Positive** | **0.183** | **0.095** | **0.271** | **<.001** |
| Calmness | Uncertain | Positive | 0.066 | -0.036 | 0.169 | 0.281 |
| **Contentment** | **Negative** | **Uncertain** | **0.175** | **0.096** | **0.255** | **<.001** |
| **Contentment** | **Negative** | **Positive** | **0.166** | **0.103** | **0.229** | **<.001** |
| Contentment | Uncertain | Positive | -0.009 | -0.082 | 0.064 | 0.953 |
| Focus | Negative | Uncertain | 0.052 | -0.024 | 0.128 | 0.238 |
| Focus | Negative | Positive | 0.047 | -0.013 | 0.107 | 0.160 |
| Focus | Uncertain | Positive | -0.005 | -0.075 | 0.065 | 0.984 |
| **Happiness** | **Negative** | **Uncertain** | **0.123** | **0.032** | **0.213** | **0.004** |
| **Happiness** | **Negative** | **Positive** | **0.143** | **0.071** | **0.215** | **<.001** |
| Happiness | Uncertain | Positive | 0.020 | -0.064 | 0.103 | 0.841 |
| **Peacefulness** | **Negative** | **Uncertain** | **0.190** | **0.101** | **0.279** | **<.001** |
| **Peacefulness** | **Negative** | **Positive** | **0.212** | **0.140** | **0.283** | **<.001** |
| Peacefulness | Uncertain | Positive | 0.022 | -0.061 | 0.104 | 0.811 |

*Note*. All reported *p*-values were adjusted for multiple comparisons.
