# Supplementary material for: Effects of self-administered binaural beats on meditative and introspective states
Source: PLoS One. 2026 Apr 1;21(4):e0335580. doi: 10.1371/journal.pone.0335580 (PMC13042839; doi:10.1371/journal.pone.0335580)
Supplement: Supplementary file 7 [file pone.0335580.s007.docx]

S7. Descriptive statistics of mood change scores alongside effect size estimates and TOST outcomes

					Change score summaries							Effect size estimates			Equivalence and one-sided t-tests	
**Study**	**Data**	**Group**	**Mood**	**n (obs)**	***Median***	***Mean***	***SD***	***SE***	***95% Low***	***95% High***	**ROPE**	***g***	***95% Low***	***95% High***	**Equivalence Test**	**One-sample t test**
Exp 1	Full	3 Hz (Delta)	Calmness	25	0.000	-0.018	0.351	0.070	-0.091	0.055	ACCEPTED	-0.050	-0.368	0.270	The equivalence test was non-significant, t(24) = 1.422, p = 8.39e-02	The null hypothesis test was non-significant, t(24) = -0.256, p = 8e-01
Exp 1	Full	3 Hz (Delta)	Contentment	25	0.077	0.040	0.254	0.051	-0.013	0.093	ACCEPTED	0.152	-0.170	0.472	The equivalence test was non-significant, t(24) = -1.533, p = 6.92e-02	The null hypothesis test was non-significant, t(24) = 0.787, p = 4.39e-01
Exp 1	Full	3 Hz (Delta)	Focus	25	0.000	0.074	0.186	0.037	0.036	0.113	ACCEPTED	0.385	0.050	0.713	The equivalence test was non-significant, t(24) = -1.179, p = 1.25e-01	The null hypothesis test was non-significant, t(24) = 1.989, p = 5.82e-02
Exp 1	Full	3 Hz (Delta)	Happiness	25	0.000	-0.036	0.211	0.042	-0.080	0.008	ACCEPTED	-0.165	-0.484	0.157	The equivalence test was significant, t(24) = 1.940, p = 3.21e-02	The null hypothesis test was non-significant, t(24) = -0.853, p = 4.02e-01
Exp 1	Full	3 Hz (Delta)	Peacefulness	25	0.067	0.054	0.212	0.042	0.010	0.098	ACCEPTED	0.247	-0.080	0.568	The equivalence test was non-significant, t(24) = -1.506, p = 7.26e-02	The null hypothesis test was non-significant, t(24) = 1.274, p = 2.15e-01
Exp 1	Full	6 Hz (Theta)	Calmness	24	0.091	0.217	0.246	0.050	0.165	0.269	REJECTED	0.853	0.461	1.230	The equivalence test was non-significant, t(23) = 1.974, p = 9.7e-01	The null hypothesis test was significant, t(23) = 4.321, p = 2.53e-04
Exp 1	Full	6 Hz (Theta)	Contentment	24	0.000	0.069	0.163	0.033	0.035	0.104	ACCEPTED	0.409	0.066	0.744	The equivalence test was non-significant, t(23) = -1.468, p = 7.78e-02	The null hypothesis test was significant, t(23) = 2.074, p = 4.95e-02
Exp 1	Full	6 Hz (Theta)	Focus	24	0.171	0.170	0.194	0.040	0.129	0.211	REJECTED	0.847	0.456	1.223	The equivalence test was non-significant, t(23) = 1.317, p = 9e-01	The null hypothesis test was significant, t(23) = 4.293, p = 2.71e-04
Exp 1	Full	6 Hz (Theta)	Happiness	24	0.000	-0.004	0.149	0.030	-0.036	0.028	ACCEPTED	-0.026	-0.350	0.299	The equivalence test was significant, t(23) = 3.743, p = 5.31e-04	The null hypothesis test was non-significant, t(23) = -0.132, p = 8.97e-01
Exp 1	Full	6 Hz (Theta)	Peacefulness	24	0.000	0.119	0.225	0.046	0.072	0.167	UNDECIDED	0.511	0.159	0.854	The equivalence test was non-significant, t(23) = 0.025, p = 5.1e-01	The null hypothesis test was significant, t(23) = 2.591, p = 1.63e-02
Exp 1	Full	9 Hz (Alpha-1)	Calmness	27	0.091	0.140	0.248	0.048	0.091	0.189	UNDECIDED	0.548	0.212	0.875	The equivalence test was non-significant, t(26) = 0.464, p = 6.77e-01	The null hypothesis test was significant, t(26) = 2.933, p = 6.92e-03
Exp 1	Full	9 Hz (Alpha-1)	Contentment	27	0.000	0.044	0.240	0.046	-0.004	0.092	ACCEPTED	0.178	-0.134	0.486	The equivalence test was non-significant, t(26) = -1.599, p = 6.1e-02	The null hypothesis test was non-significant, t(26) = 0.953, p = 3.5e-01
Exp 1	Full	9 Hz (Alpha-1)	Focus	27	0.000	0.041	0.177	0.034	0.006	0.076	ACCEPTED	0.225	-0.089	0.534	The equivalence test was significant, t(26) = -2.256, p = 1.63e-02	The null hypothesis test was non-significant, t(26) = 1.204, p = 2.4e-01
Exp 1	Full	9 Hz (Alpha-1)	Happiness	27	0.000	-0.040	0.193	0.037	-0.078	-0.002	ACCEPTED	-0.201	-0.510	0.111	The equivalence test was significant, t(26) = 2.096, p = 2.3e-02	The null hypothesis test was non-significant, t(26) = -1.077, p = 2.91e-01
Exp 1	Full	9 Hz (Alpha-1)	Peacefulness	27	0.000	0.081	0.227	0.044	0.036	0.126	UNDECIDED	0.346	0.026	0.660	The equivalence test was non-significant, t(26) = -0.844, p = 2.03e-01	The null hypothesis test was non-significant, t(26) = 1.854, p = 7.51e-02
Exp 1	Full	12 Hz (Alpha-2)	Calmness	25	0.143	0.181	0.245	0.049	0.131	0.232	REJECTED	0.715	0.348	1.070	The equivalence test was non-significant, t(24) = 1.289, p = 8.95e-01	The null hypothesis test was significant, t(24) = 3.694, p = 1.14e-03
Exp 1	Full	12 Hz (Alpha-2)	Contentment	25	0.000	0.034	0.237	0.047	-0.015	0.083	ACCEPTED	0.139	-0.183	0.458	The equivalence test was significant, t(24) = -1.769, p = 4.48e-02	The null hypothesis test was non-significant, t(24) = 0.717, p = 4.8e-01
Exp 1	Full	12 Hz (Alpha-2)	Focus	25	0.000	0.080	0.214	0.043	0.036	0.124	UNDECIDED	0.362	0.029	0.688	The equivalence test was non-significant, t(24) = -0.884, p = 1.93e-01	The null hypothesis test was non-significant, t(24) = 1.869, p = 7.39e-02
Exp 1	Full	12 Hz (Alpha-2)	Happiness	25	0.000	0.011	0.106	0.021	-0.011	0.033	ACCEPTED	0.100	-0.220	0.419	The equivalence test was significant, t(24) = -5.040, p = 1.88e-05	The null hypothesis test was non-significant, t(24) = 0.519, p = 6.09e-01
Exp 1	Full	12 Hz (Alpha-2)	Peacefulness	25	0.000	0.068	0.180	0.036	0.031	0.105	ACCEPTED	0.366	0.032	0.692	The equivalence test was non-significant, t(24) = -1.385, p = 8.94e-02	The null hypothesis test was non-significant, t(24) = 1.889, p = 7.1e-02
Exp 1	No-Outliers	3 Hz (Delta)	Calmness	18	0.000	0.001	0.135	0.032	-0.033	0.035	ACCEPTED	0.007	-0.363	0.377	The equivalence test was significant, t(17) = -3.672, p = 9.44e-04	The null hypothesis test was non-significant, t(17) = 0.0314, p = 9.75e-01
Exp 1	No-Outliers	3 Hz (Delta)	Contentment	23	0.111	0.087	0.203	0.042	0.043	0.131	UNDECIDED	0.414	0.063	0.756	The equivalence test was non-significant, t(22) = -0.729, p = 2.37e-01	The null hypothesis test was non-significant, t(22) = 2.055, p = 5.19e-02
Exp 1	No-Outliers	3 Hz (Delta)	Focus	24	0.000	0.056	0.167	0.034	0.021	0.091	ACCEPTED	0.324	-0.013	0.655	The equivalence test was significant, t(23) = -1.814, p = 4.13e-02	The null hypothesis test was non-significant, t(23) = 1.643, p = 1.14e-01
Exp 1	No-Outliers	3 Hz (Delta)	Happiness	22	-0.033	-0.055	0.147	0.031	-0.088	-0.023	ACCEPTED	-0.361	-0.707	-0.006	The equivalence test was significant, t(21) = 2.005, p = 2.9e-02	The null hypothesis test was non-significant, t(21) = -1.755, p = 9.39e-02
Exp 1	No-Outliers	3 Hz (Delta)	Peacefulness	23	0.000	0.015	0.172	0.036	-0.022	0.052	ACCEPTED	0.084	-0.249	0.415	The equivalence test was significant, t(22) = -2.868, p = 4.47e-03	The null hypothesis test was non-significant, t(22) = 0.418, p = 6.8e-01
Exp 1	No-Outliers	6 Hz (Theta)	Calmness	22	0.045	0.176	0.213	0.045	0.129	0.224	REJECTED	0.796	0.395	1.182	The equivalence test was non-significant, t(21) = 1.280, p = 8.93e-01	The null hypothesis test was significant, t(21) = 3.876, p = 8.74e-04
Exp 1	No-Outliers	6 Hz (Theta)	Contentment	24	0.000	0.069	0.163	0.033	0.035	0.104	ACCEPTED	0.409	0.066	0.744	The equivalence test was non-significant, t(23) = -1.468, p = 7.78e-02	The null hypothesis test was significant, t(23) = 2.074, p = 4.95e-02
Exp 1	No-Outliers	6 Hz (Theta)	Focus	23	0.200	0.189	0.174	0.036	0.152	0.227	REJECTED	1.049	0.619	1.460	The equivalence test was non-significant, t(22) = 1.961, p = 9.69e-01	The null hypothesis test was significant, t(22) = 5.209, p = 3.18e-05
Exp 1	No-Outliers	6 Hz (Theta)	Happiness	23	0.000	0.018	0.108	0.023	-0.006	0.042	ACCEPTED	0.161	-0.174	0.493	The equivalence test was significant, t(22) = -4.434, p = 1.04e-04	The null hypothesis test was non-significant, t(22) = 0.799, p = 4.33e-01
Exp 1	No-Outliers	6 Hz (Theta)	Peacefulness	21	0.000	0.099	0.170	0.037	0.061	0.138	UNDECIDED	0.560	0.179	0.928	The equivalence test was non-significant, t(20) = -0.508, p = 3.08e-01	The null hypothesis test was significant, t(20) = 2.669, p = 1.48e-02
Exp 1	No-Outliers	9 Hz (Alpha-1)	Calmness	25	0.091	0.154	0.175	0.035	0.118	0.190	REJECTED	0.852	0.468	1.222	The equivalence test was non-significant, t(24) = 1.033, p = 8.44e-01	The null hypothesis test was significant, t(24) = 4.400, p = 1.91e-04
Exp 1	No-Outliers	9 Hz (Alpha-1)	Contentment	23	0.000	0.014	0.134	0.028	-0.015	0.043	ACCEPTED	0.101	-0.232	0.432	The equivalence test was significant, t(22) = -3.717, p = 6e-04	The null hypothesis test was non-significant, t(22) = 0.501, p = 6.21e-01
Exp 1	No-Outliers	9 Hz (Alpha-1)	Focus	26	0.000	0.024	0.155	0.030	-0.008	0.056	ACCEPTED	0.150	-0.166	0.463	The equivalence test was significant, t(25) = -3.087, p = 2.44e-03	The null hypothesis test was non-significant, t(25) = 0.790, p = 4.37e-01
Exp 1	No-Outliers	9 Hz (Alpha-1)	Happiness	25	0.000	-0.003	0.146	0.029	-0.033	0.027	ACCEPTED	-0.020	-0.338	0.299	The equivalence test was significant, t(24) = 3.933, p = 3.12e-04	The null hypothesis test was non-significant, t(24) = -0.103, p = 9.19e-01
Exp 1	No-Outliers	9 Hz (Alpha-1)	Peacefulness	25	0.000	0.079	0.137	0.027	0.051	0.108	ACCEPTED	0.558	0.208	0.898	The equivalence test was non-significant, t(24) = -1.418, p = 8.45e-02	The null hypothesis test was significant, t(24) = 2.883, p = 8.18e-03
Exp 1	No-Outliers	12 Hz (Alpha-2)	Calmness	23	0.143	0.179	0.196	0.041	0.137	0.222	REJECTED	0.882	0.477	1.270	The equivalence test was non-significant, t(22) = 1.496, p = 9.26e-01	The null hypothesis test was significant, t(22) = 4.380, p = 2.39e-04
Exp 1	No-Outliers	12 Hz (Alpha-2)	Contentment	23	0.000	0.042	0.146	0.030	0.011	0.074	ACCEPTED	0.278	-0.063	0.613	The equivalence test was significant, t(22) = -2.492, p = 1.04e-02	The null hypothesis test was non-significant, t(22) = 1.380, p = 1.82e-01
Exp 1	No-Outliers	12 Hz (Alpha-2)	Focus	24	0.000	0.051	0.160	0.033	0.017	0.085	ACCEPTED	0.308	-0.028	0.638	The equivalence test was significant, t(23) = -2.047, p = 2.61e-02	The null hypothesis test was non-significant, t(23) = 1.562, p = 1.32e-01
Exp 1	No-Outliers	12 Hz (Alpha-2)	Happiness	25	0.000	0.011	0.106	0.021	-0.011	0.033	ACCEPTED	0.100	-0.220	0.419	The equivalence test was significant, t(24) = -5.040, p = 1.88e-05	The null hypothesis test was non-significant, t(24) = 0.519, p = 6.09e-01
Exp 1	No-Outliers	12 Hz (Alpha-2)	Peacefulness	24	0.000	0.047	0.152	0.031	0.015	0.079	ACCEPTED	0.299	-0.037	0.628	The equivalence test was significant, t(23) = -2.284, p = 1.6e-02	The null hypothesis test was non-significant, t(23) = 1.515, p = 1.43e-01
Exp 2	Full	BB + Pink Noise	Calmness	34	0.250	0.285	0.277	0.047	0.237	0.334	REJECTED	1.005	0.657	1.341	The equivalence test was non-significant, t(33) = 3.519, p = 9.99e-01	The null hypothesis test was significant, t(33) = 5.999, p = 9.67e-07
Exp 2	Full	BB + Pink Noise	Contentment	34	0.000	0.018	0.228	0.039	-0.022	0.058	ACCEPTED	0.077	-0.200	0.353	The equivalence test was significant, t(33) = -2.554, p = 7.73e-03	The null hypothesis test was non-significant, t(33) = 0.460, p = 6.48e-01
Exp 2	Full	BB + Pink Noise	Focus	34	0.134	0.184	0.188	0.032	0.152	0.217	REJECTED	0.956	0.614	1.287	The equivalence test was non-significant, t(33) = 2.052, p = 9.76e-01	The null hypothesis test was significant, t(33) = 5.707, p = 2.29e-06
Exp 2	Full	BB + Pink Noise	Happiness	34	0.063	0.000	0.240	0.041	-0.042	0.042	ACCEPTED	0.000	-0.276	0.276	The equivalence test was significant, t(33) = -2.863, p = 3.62e-03	The null hypothesis test was non-significant, t(33) = 0.000, p = 1e+00
Exp 2	Full	BB + Pink Noise	Peacefulness	34	0.059	0.059	0.247	0.042	0.016	0.102	ACCEPTED	0.233	-0.048	0.511	The equivalence test was non-significant, t(33) = -1.389, p = 8.7e-02	The null hypothesis test was non-significant, t(33) = 1.393, p = 1.73e-01
Exp 2	Full	BB Only	Calmness	34	0.200	0.238	0.314	0.054	0.184	0.293	REJECTED	0.741	0.422	1.049	The equivalence test was non-significant, t(33) = 2.231, p = 9.84e-01	The null hypothesis test was significant, t(33) = 4.420, p = 1.01e-04
Exp 2	Full	BB Only	Contentment	34	0.000	0.052	0.166	0.028	0.023	0.081	ACCEPTED	0.306	0.021	0.586	The equivalence test was significant, t(33) = -2.313, p = 1.35e-02	The null hypothesis test was non-significant, t(33) = 1.827, p = 7.68e-02
Exp 2	Full	BB Only	Focus	34	0.125	0.157	0.168	0.029	0.128	0.187	REJECTED	0.913	0.576	1.239	The equivalence test was non-significant, t(33) = 1.359, p = 9.08e-01	The null hypothesis test was significant, t(33) = 5.449, p = 4.9e-06
Exp 2	Full	BB Only	Happiness	34	0.000	-0.126	0.231	0.040	-0.167	-0.086	UNDECIDED	-0.533	-0.825	-0.233	The equivalence test was non-significant, t(33) = -0.206, p = 5.81e-01	The null hypothesis test was significant, t(33) = -3.181, p = 3.19e-03
Exp 2	Full	BB Only	Peacefulness	34	0.000	0.044	0.235	0.040	0.003	0.085	ACCEPTED	0.183	-0.097	0.460	The equivalence test was significant, t(33) = -1.832, p = 3.8e-02	The null hypothesis test was non-significant, t(33) = 1.092, p = 2.83e-01
Exp 2	Full	Pink Noise only	Calmness	28	0.029	0.057	0.292	0.055	0.001	0.114	ACCEPTED	0.190	-0.117	0.493	The equivalence test was non-significant, t(27) = -1.103, p = 1.4e-01	The null hypothesis test was non-significant, t(27) = 1.033, p = 3.11e-01
Exp 2	Full	Pink Noise only	Contentment	28	0.000	-0.015	0.235	0.044	-0.061	0.031	ACCEPTED	-0.062	-0.364	0.241	The equivalence test was significant, t(27) = 2.316, p = 1.42e-02	The null hypothesis test was non-significant, t(27) = -0.338, p = 7.38e-01
Exp 2	Full	Pink Noise only	Focus	28	0.000	-0.037	0.233	0.044	-0.082	0.008	ACCEPTED	-0.154	-0.457	0.151	The equivalence test was significant, t(27) = 1.836, p = 3.87e-02	The null hypothesis test was non-significant, t(27) = -0.840, p = 4.08e-01
Exp 2	Full	Pink Noise only	Happiness	28	0.000	-0.009	0.286	0.054	-0.065	0.047	ACCEPTED	-0.031	-0.332	0.272	The equivalence test was significant, t(27) = 2.014, p = 2.7e-02	The null hypothesis test was non-significant, t(27) = -0.167, p = 8.69e-01
Exp 2	Full	Pink Noise only	Peacefulness	28	-0.029	-0.065	0.336	0.063	-0.130	0.000	UNDECIDED	-0.188	-0.491	0.119	The equivalence test was non-significant, t(27) = 0.832, p = 2.06e-01	The null hypothesis test was non-significant, t(27) = -1.024, p = 3.15e-01
Exp 2	Full	Silence	Calmness	22	0.000	0.011	0.324	0.069	-0.061	0.083	ACCEPTED	0.033	-0.306	0.370	The equivalence test was non-significant, t(21) = -1.547, p = 6.84e-02	The null hypothesis test was non-significant, t(21) = 0.159, p = 8.75e-01
Exp 2	Full	Silence	Contentment	22	0.000	-0.037	0.317	0.068	-0.108	0.034	ACCEPTED	-0.112	-0.450	0.228	The equivalence test was non-significant, t(21) = 1.196, p = 1.22e-01	The null hypothesis test was non-significant, t(21) = -0.547, p = 5.9e-01
Exp 2	Full	Silence	Focus	22	0.000	0.041	0.185	0.039	0.000	0.082	ACCEPTED	0.214	-0.131	0.553	The equivalence test was significant, t(21) = -1.949, p = 3.24e-02	The null hypothesis test was non-significant, t(21) = 1.039, p = 3.1e-01
Exp 2	Full	Silence	Happiness	22	0.000	-0.065	0.280	0.060	-0.127	-0.003	UNDECIDED	-0.224	-0.564	0.122	The equivalence test was non-significant, t(21) = 0.885, p = 1.93e-01	The null hypothesis test was non-significant, t(21) = -1.089, p = 2.89e-01
Exp 2	Full	Silence	Peacefulness	22	0.000	-0.046	0.222	0.047	-0.095	0.003	ACCEPTED	-0.200	-0.539	0.144	The equivalence test was non-significant, t(21) = 1.518, p = 7.19e-02	The null hypothesis test was non-significant, t(21) = -0.972, p = 3.42e-01
Exp 2	No-Outliers	BB + Pink Noise	Calmness	14	0.261	0.233	0.164	0.044	0.186	0.281	REJECTED	1.337	0.725	1.914	The equivalence test was non-significant, t(13) = 2.627, p = 9.9e-01	The null hypothesis test was significant, t(13) = 5.316, p = 1.4e-04
Exp 2	No-Outliers	BB + Pink Noise	Contentment	27	0.000	0.036	0.126	0.024	0.011	0.061	ACCEPTED	0.277	-0.039	0.588	The equivalence test was significant, t(26) = -3.376, p = 1.16e-03	The null hypothesis test was non-significant, t(26) = 1.485, p = 1.5e-01
Exp 2	No-Outliers	BB + Pink Noise	Focus	18	0.200	0.218	0.141	0.033	0.183	0.253	REJECTED	1.477	0.907	2.017	The equivalence test was non-significant, t(17) = 3.013, p = 9.96e-01	The null hypothesis test was significant, t(17) = 6.560, p = 4.86e-06
Exp 2	No-Outliers	BB + Pink Noise	Happiness	28	0.067	0.040	0.101	0.019	0.021	0.060	ACCEPTED	0.385	0.067	0.696	The equivalence test was significant, t(27) = -4.079, p = 1.8e-04	The null hypothesis test was significant, t(27) = 2.096, p = 4.56e-02
Exp 2	No-Outliers	BB + Pink Noise	Peacefulness	26	0.059	0.057	0.117	0.023	0.034	0.081	ACCEPTED	0.472	0.137	0.799	The equivalence test was significant, t(25) = -2.652, p = 6.85e-03	The null hypothesis test was significant, t(25) = 2.484, p = 2e-02
Exp 2	No-Outliers	BB Only	Calmness	13	0.200	0.211	0.131	0.036	0.172	0.251	REJECTED	1.508	0.829	2.145	The equivalence test was non-significant, t(12) = 2.564, p = 9.88e-01	The null hypothesis test was significant, t(12) = 5.807, p = 8.38e-05
Exp 2	No-Outliers	BB Only	Contentment	25	0.000	0.057	0.125	0.025	0.031	0.083	ACCEPTED	0.442	0.102	0.772	The equivalence test was significant, t(24) = -2.434, p = 1.14e-02	The null hypothesis test was significant, t(24) = 2.280, p = 3.18e-02
Exp 2	No-Outliers	BB Only	Focus	27	0.125	0.143	0.139	0.027	0.116	0.171	UNDECIDED	0.999	0.609	1.373	The equivalence test was non-significant, t(26) = 0.940, p = 8.22e-01	The null hypothesis test was significant, t(26) = 5.346, p = 1.35e-05
Exp 2	No-Outliers	BB Only	Happiness	16	0.000	-0.102	0.152	0.038	-0.143	-0.062	UNDECIDED	-0.637	-1.061	-0.194	The equivalence test was non-significant, t(15) = 0.417, p = 3.41e-01	The null hypothesis test was significant, t(15) = -2.684, p = 1.7e-02
Exp 2	No-Outliers	BB Only	Peacefulness	28	0.000	0.060	0.114	0.022	0.038	0.082	ACCEPTED	0.512	0.184	0.830	The equivalence test was significant, t(27) = -2.685, p = 6.12e-03	The null hypothesis test was significant, t(27) = 2.785, p = 9.66e-03
Exp 2	No-Outliers	Pink Noise only	Calmness	18	0.000	0.092	0.171	0.040	0.050	0.135	UNDECIDED	0.514	0.110	0.904	The equivalence test was non-significant, t(17) = -0.641, p = 2.65e-01	The null hypothesis test was significant, t(17) = 2.283, p = 3.56e-02
Exp 2	No-Outliers	Pink Noise only	Contentment	21	0.000	-0.011	0.140	0.031	-0.043	0.021	ACCEPTED	-0.076	-0.420	0.271	The equivalence test was significant, t(20) = 3.498, p = 1.13e-03	The null hypothesis test was non-significant, t(20) = -0.360, p = 7.23e-01
Exp 2	No-Outliers	Pink Noise only	Focus	20	0.000	-0.010	0.181	0.040	-0.053	0.033	ACCEPTED	-0.053	-0.406	0.301	The equivalence test was significant, t(19) = 2.665, p = 7.65e-03	The null hypothesis test was non-significant, t(19) = -0.247, p = 8.07e-01
Exp 2	No-Outliers	Pink Noise only	Happiness	19	0.000	-0.006	0.137	0.031	-0.039	0.027	ACCEPTED	-0.042	-0.403	0.320	The equivalence test was significant, t(18) = 3.559, p = 1.12e-03	The null hypothesis test was non-significant, t(18) = -0.191, p = 8.51e-01
Exp 2	No-Outliers	Pink Noise only	Peacefulness	14	-0.029	-0.069	0.096	0.026	-0.097	-0.042	ACCEPTED	-0.676	-1.132	-0.198	The equivalence test was significant, t(13) = 1.904, p = 3.96e-02	The null hypothesis test was significant, t(13) = -2.689, p = 1.86e-02
Exp 2	No-Outliers	Silence	Calmness	12	0.000	0.022	0.103	0.030	-0.011	0.055	ACCEPTED	0.199	-0.253	0.641	The equivalence test was significant, t(11) = -3.224, p = 4.05e-03	The null hypothesis test was non-significant, t(11) = 0.740, p = 4.75e-01
Exp 2	No-Outliers	Silence	Contentment	14	0.000	0.000	0.122	0.033	-0.035	0.035	ACCEPTED	0.000	-0.414	0.414	The equivalence test was significant, t(13) = -3.614, p = 1.57e-03	The null hypothesis test was non-significant, t(13) = 0.000, p = 1e+00
Exp 2	No-Outliers	Silence	Focus	17	0.000	0.016	0.136	0.033	-0.019	0.051	ACCEPTED	0.112	-0.271	0.492	The equivalence test was significant, t(16) = -3.088, p = 3.53e-03	The null hypothesis test was non-significant, t(16) = 0.485, p = 6.34e-01
Exp 2	No-Outliers	Silence	Happiness	16	0.000	0.019	0.112	0.028	-0.011	0.049	ACCEPTED	0.161	-0.235	0.552	The equivalence test was significant, t(15) = -3.530, p = 1.51e-03	The null hypothesis test was non-significant, t(15) = 0.679, p = 5.08e-01
Exp 2	No-Outliers	Silence	Peacefulness	19	0.000	-0.010	0.139	0.032	-0.044	0.024	ACCEPTED	-0.069	-0.430	0.294	The equivalence test was significant, t(18) = 3.382, p = 1.66e-03	The null hypothesis test was non-significant, t(18) = -0.314, p = 7.57e-01
