# Supplementary material for: Effects of self-administered binaural beats on meditative and introspective states
Source: PLoS One. 2026 Apr 1;21(4):e0335580. doi: 10.1371/journal.pone.0335580 (PMC13042839; doi:10.1371/journal.pone.0335580)
Supplement: Attachment — Submitted filename: bb2_reviewer_response.docx [file pone.0335580.s012.docx]

**Response to Reviewers**

[Author responses in red]

Dear Dr. Amd,

Thank you for submitting your manuscript to PLOS ONE. After careful consideration, we feel that it has merit but does not fully meet PLOS ONE’s publication criteria as it currently stands. Therefore, we invite you to submit a revised version of the manuscript that addresses the points raised during the review process. Please submit your revised manuscript by Jan 13 2026 11:59PM. If you will need more time than this to complete your revisions, please reply to this message or contact the journal office at plosone@plos.org. When you're ready to submit your revision, log on to https://www.editorialmanager.com/pone/ and select the 'Submissions Needing Revision' folder to locate your manuscript file. Please include the following items when submitting your revised manuscript:

A rebuttal letter that responds to each point raised by the academic editor and reviewer(s). You should upload this letter as a separate file labeled 'Response to Reviewers'.

Response: Provided below

A marked-up copy of your manuscript that highlights changes made to the original version. You should upload this as a separate file labeled 'Revised Manuscript with Track Changes'.

Response: Attached

An unmarked version of your revised paper without tracked changes. You should upload this as a separate file labeled 'Manuscript'.

Response: Attached

If you would like to make changes to your financial disclosure, please include your updated statement in your cover letter. Guidelines for resubmitting your figure files are available below the reviewer comments at the end of this letter.

Response: A Financial Disclosure statement has been included with the updated cover letter.

If applicable, we recommend that you deposit your laboratory protocols in protocols.io to enhance the reproducibility of your results. Protocols.io assigns your protocol its own identifier (DOI) so that it can be cited independently in the future. For instructions see: https://journals.plos.org/plosone/s/submission-guidelines#loc-laboratory-protocols. Additionally, PLOS ONE offers an option for publishing peer-reviewed Lab Protocol articles, which describe protocols hosted on protocols.io. Read more information on sharing protocols at https://plos.org/protocols?utm_medium=editorial-email&utm_source=authorletters&utm_campaign=protocols.

We look forward to receiving your revised manuscript.

Kind regards,

Francesco Bossi

Academic Editor

PLOS ONE

Journal Requirements:

When submitting your revision, we need you to address these additional requirements.

1.Please ensure that your manuscript meets PLOS ONE's style requirements, including those for file naming. The PLOS ONE style templates can be found at

<https://journals.plos.org/plosone/s/file?id=wjVg/PLOSOne_formatting_sample_main_body.pdf> and <https://journals.plos.org/plosone/s/file?id=ba62/PLOSOne_formatting_sample_title_authors_affiliations.pdf>

2. We note that the grant information you provided in the ‘Funding Information’ and ‘Financial Disclosure’ sections do not match. When you resubmit, please ensure that you provide the correct grant numbers for the awards you received for your study in the ‘Funding Information’ section.

Response: The correct grant number (AURC 02/2025/1.1.1) has been provided in the ‘Funding Information’ and ‘Financial Disclosure’ sections.

3. Thank you for stating the following financial disclosure: “The current research was funded by an internal grant (AURC 02/2025/1.1.1) from the USP research office to the author.” Please state what role the funders took in the study. If the funders had no role, please state: "The funders had no role in study design, data collection and analysis, decision to publish, or preparation of the manuscript." If this statement is not correct you must amend it as needed. *Please include this amended Role of Funder statement in your cover letter; we will change the online submission form on your behalf.

Response: The following statement has been included in the financial disclosure section: "The funders had no role in study design, data collection and analysis, decision to publish, or preparation of the manuscript."

4. Please note that your Data Availability Statement is currently missing the repository name. If your manuscript is accepted for publication, you will be asked to provide these details on a very short timeline. We therefore suggest that you provide this information now, though we will not hold up the peer review process if you are unable.

Response: The GitHub repository containing all data files (<https://github.com/micahamd/BB2-Files>) have been included in the Data Availability Statement, and under Study Materials in the revised manuscript.

5. When completing the data availability statement of the submission form, you indicated that you will make your data available on acceptance. We strongly recommend all authors decide on a data sharing plan before acceptance, as the process can be lengthy and hold up publication timelines. Please note that, though access restrictions are acceptable now, your entire data will need to be made freely accessible if your manuscript is accepted for publication. This policy applies to all data except where public deposition would breach compliance with the protocol approved by your research ethics board. If you are unable to adhere to our open data policy, please kindly revise your statement to explain your reasoning and we will seek the editor's input on an exemption. Please be assured that, once you have provided your new statement, the assessment of your exemption will not hold up the peer review process.

Response: All data and scripts are available in a GitHub repository (<https://github.com/micahamd/BB2-Files>)

6.If the reviewer comments include a recommendation to cite specific previously published works, please review and evaluate these publications to determine whether they are relevant and should be cited. There is no requirement to cite these works unless the editor has indicated otherwise.

Response: Understood.

7. Please review your reference list to ensure that it is complete and correct. If you have cited papers that have been retracted, please include the rationale for doing so in the manuscript text, or remove these references and replace them with relevant current references. Any changes to the reference list should be mentioned in the rebuttal letter that accompanies your revised manuscript. If you need to cite a retracted article, indicate the article’s retracted status in the References list and also include a citation and full reference for the retraction notice.

Response: The reference list has been reviewed. No retracted papers are cited.

Additional Editor Comments:

Both Reviewers found it appropriate to ask for minor revisions for the current manuscript. With thorough adjustments and changes, the manuscript can be made available for publication.

Response: We are grateful for the constructive comments and suggestions. All requested edits have been thoroughly administered, which has significantly strengthened the manuscript.

=======

[Note: HTML markup is below. Please do not edit.]

Reviewer's Responses to Questions

Comments to the Author

1. Is the manuscript technically sound, and do the data support the conclusions?

The manuscript must describe a technically sound piece of scientific research with data that supports the conclusions. Experiments must have been conducted rigorously, with appropriate controls, replication, and sample sizes. The conclusions must be drawn appropriately based on the data presented.

Reviewer #1: Yes

Reviewer #2: Yes

2. Has the statistical analysis been performed appropriately and rigorously?

Reviewer #1: Yes

Reviewer #2: Yes

3. Have the authors made all data underlying the findings in their manuscript fully available?

The PLOS Data policy requires authors to make all data underlying the findings described in their manuscript fully available without restriction, with rare exception (please refer to the Data Availability Statement in the manuscript PDF file). The data should be provided as part of the manuscript or its supporting information, or deposited to a public repository. For example, in addition to summary statistics, the data points behind means, medians and variance measures should be available. If there are restrictions on publicly sharing data—e.g. participant privacy or use of data from a third party—those must be specified.

Reviewer #1: Yes

Reviewer #2: Yes

4. Is the manuscript presented in an intelligible fashion and written in standard English?

PLOS ONE does not copyedit accepted manuscripts, so the language in submitted articles must be clear, correct, and unambiguous. Any typographical or grammatical errors should be corrected at revision, so please note any specific errors here.

Reviewer #1: Yes

Reviewer #2: Yes

5. **Review Comments to the Author**

Please use the space provided to explain your answers to the questions above. You may also include additional comments for the author, including concerns about dual publication, research ethics, or publication ethics. (Please upload your review as an attachment if it exceeds 20,000 characters)

Response: All requested changes have been addressed.

*Reviewer #1:* The submitted manuscript presents a comprehensive investigation into the behavioral effects of brief, self-administered theta-frequency (6 Hz) binaural beats (BBs) on mood states, specifically calmness and focus, in undergraduate students. The authors report two complementary studies: Study 1 compares frequency-specific effects across delta, theta, and alpha ranges, while Study 2 evaluates the effects of 6 Hz BBs against control conditions (pink noise, silence) and includes qualitative introspective measures. The methodology leverages an online self-administration protocol via a custom Web Audio API application, providing an ecologically valid and scalable approach. Overall, the manuscript is thorough, methodologically rigorous, and addresses a timely question in the field of auditory brainwave modulation. The writing is detailed, and the analyses are statistically sophisticated, incorporating both traditional hypothesis testing and equivalence testing.

Response: Thank you for your valuable comments. We hope the revisions address your concerns satisfactorily.

In what follows, my comments to the submitted work:

The introduction is dense and highly technical. Simplifying some passages or moving detailed neurophysiological explanations to supplementary material could improve readability.

Response: The neurophysiological explanations have been reduced by a third in the Introduction on page 4 to avoid unnecessary detail.

1. While the literature review is thorough, the discussion of prior inconsistent findings could be more concise.

Response: Prior literature discussing inconsistent outcomes have been presented more concisely on page 4: "...a systematic review by (5) questioned the validity of BEH given inconsistent empirical outcomes, though these were likened to stem from the methodological heterogeneity in EEG measurement approaches used, varying operational definitions of what ‘entrainment’ entails (auditory steady-state responses versus oscillatory power changes), and inconsistent control procedures across studies."

2. Consider briefly justifying why 3 Hz and 12 Hz were included, as readers may question their relevance to delta and alpha ranges.

Response: Justifications for the frequency conditions (checking for ramping effects, exploring frequency-specific mood effects along conventionally defined bands) have been provided on page 8: "The comparison of physically equidistant frequencies allowed exploring for any systemic relationships between progressive lowering (or increasing) BB frequencies on relaxation or any other mood states (ramping effects). Additionally, the non-theta BB frequency conditions align with well-established delta and alpha oscillatory activity bands, which have been variably associated with different mood states in prior research (4, 5). Comparing across these conditions would help identify whether (any) significant modulations across mood states were particular to a given frequency."

3. No screening was reported for hearing deficits, neurological conditions, or use of medications that could affect auditory perception; this should be acknowledged as a limitation.

Response: The following statement has been included under Limitations section on p. 31: "...the non-supervised protocol did not incorporate screening for the presence of specific hearing deficits, neurological conditions, or medication histories that could affect auditory perception. We acknowledge that these uncontrolled variables (environmental noise, distractions, headphone quality, individual volume preferences, auditory perceptual capacity) introduce measurement error that future protocols should address.”

4. Participant-adjusted volume introduces variability; discuss potential effects on mood measures.

Response: The limitations' section on p. 30 now includes the following: "Inspection of negative sentiments (S6) revealed recurring references to auditory characteristics (e.g., 'The sound was too loud,' 'I felt annoyance at the audio'), highlighting the variability introduced by participant-adjusted volume on outcomes, in spite of the pre-BB audio calibration phase implemented to mitigate this issue. While participant-adjusted volume aimed to enhance ecological validity, it introduced a potential confound in mood measurement that future work should address through standardized protocols or measured covariates...".

5. Clarify rationale for frequency selection and exposure duration in the Methods for ease of understanding.

Response: The rationale for frequency selection is clarified on p. 6: " This work focuses on theta BBs given its increasingly acknowledged role as a functional, and possibly manipulable, bio-marker of mood states associated with cognitive and emotional self-regulation (6, 8)." The rationale for exposure duration is clarified on p. 7: "The selection of a five-minute exposure duration, rather than the ten to thirty minutes as reported in earlier studies (6, 7, 8, 11), was motivated by considerations to minimize potential participant harm while ensuring minimal conditions for entrainment were met…"

6. The Results section is highly technical and dense; providing brief interpretive summaries after each major analysis could improve accessibility.

Response: Brief interpretive summary statements have been included at the end of each Results section on pages 24-28. These include:

- Study 1 ANOVAs on p.24: "These results demonstrate that the 6 Hz frequency condition produced more substantial mood improvements than the 3 Hz condition, particularly for focus and calmness, with happiness remaining relatively unaffected across conditions."
- Study 1 TOSTs on p.26: "Equivalence tests confirmed that 6 Hz and 12 Hz frequencies produced practically meaningful increases in calmness, with 6 Hz additionally increasing focus, at levels that substantially exceed ROPE thresholds for practical significance. Removing outliers revealed that 9 Hz also produced a meaningful calmness effect previously masked by data variability."
- Study 2 ANOVAs on p.27: "These results demonstrate that conditions containing theta BBs, either alone or combined with pink noise, produced significantly greater mood improvements than conditions without binaural beats (pink noise alone or silence). Calmness emerged as the most responsive mood state, showing the largest changes and driving the interaction effect, particularly when BBs were combined with pink noise."
- Study 2 TOSTs on p.28: "Equivalence tests confirmed that 6 Hz BBs, whether presented alone or combined with pink noise, produced practically meaningful increases in calmness and focus. While participants also reported increased happiness in the binaural beats only and silence conditions, and increased calmness and peacefulness with pink noise alone, these effects were either too small or too variable to confidently reject practical equivalence."
- Study 2 Sentiment Analyses on p. 30: " Quantitative analysis of qualitative sentiments indicated significantly higher positive subjective experiences and expressed willingness to repeat the intervention across both BB conditions. Pink noise alone produced polarized sentiments – participants either found it beneficial or aversive, with few in-between responses. The silence condition generated no clear preference pattern, suggesting participants found it neither particularly engaging nor problematic. Subjective sentiments significantly co-varied with most moods other than focus, implying negative introspective sentiments co-varied significantly with negative mood shifts."

7. The connection between quantitative results and qualitative sentiment data could be more explicitly discussed.

Response: The connection between quantitative results and qualitative sentiment data have been examined more thoroughly in a new section under the Results on p.29: "To directly examine the relationship between quantitative mood changes and qualitative sentiments, five within-subjects ANOVA were run per mood to explore whether coded sentiments covaried with change scores (Figure 5). Significant omnibus effects were found ...". A new Figure 5 and a new supplementary table (S8 - post-hoc tests) have been included in to accompany the extended analysis.

8. It is unclear whether corrections for multiple comparisons were applied across post-hoc and equivalence tests

Response: The fdr-correction for multiple comparisons for post-hoc tests has been declared on p. 24. For equivalence tests, no corrections for multiple comparisons were used since, for an effect to reject practical equivalence viz. a meaningful effect, both one-sided tests must reach significance, which inherently protects against false positives. Additionally, for observed effects that rejected equivalence in the reported study, *p*-values were sufficiently small to meet even Bonferroni corrected thresholds if considered. The following has been included on p. 26: "No corrections for multiple comparisons were applied to equivalence tests, as TOST procedures test for negligible effects rather than differences, and the requirement for two one-sided tests to reach significance provides inherent Type I error control (14). Additionally, all effects rejecting practical equivalence (Table 1) exhibited *p*-values well below conventional thresholds (all *p*'s < .001), suggesting our conclusions would remain robust even under conservative correction procedures."

9. I would suggest including a summary figure illustrating key frequency/condition effects across moods.

Response: A new Figure S9 has been included in the supplementary files to summarily convey mood effects across independent participant groups for both studies using crossbar plots. This has been included as a supplementary file figure it summarizes information already present across Figures 2 and 3 in the main manuscript.

Also, consider a concise “take-home message” at the end of each study’s results to highlight the main findings.

Response: Figure 3's caption in the main manuscript now includes the following take-home message: "Exposure to 6 Hz theta BBs, whether presented on their own or with a pink noise background, produced practical and non-negligible increases in self-reported calmness and focus." Additional take-home messages have been added at the end of each study’s results to summarize main findings.

10. Emphasize more strongly that conclusions regarding neural entrainment remain speculative, as no direct physiological measures were collected.

Response: The revised Discussion emphasizes this on p. 34: "The selective enhancement of regulatory mood states (calmness, focus) rather than hedonic states (happiness, contentment) aligns with established literature linking theta rhythms to frontal executive control networks (6, 25). However, it is important to emphasize that without direct physiological measures (e.g., EEG, fMRI), conclusions regarding neural entrainment remain speculative. The behavioral data reported here cannot inform claims about underlying neural mechanisms, which is a limitation shared by approximately 80% of published BB research that also lack neural measurements (26, 27).”

*Reviewer #2*: The paper investigates the effects of self-administered binaural beats on subjective mood evaluations. The dataset is extensive, and the statistical analyses appear rigorous and appropriate. However, the study relies exclusively on qualitative and self-reported measures, which limits the strength of the conclusions.

Response: Thank you for your valuable comments. We have explicitly acknowledged the lack of neurological measures as a limitation on p. 34.

Specific comments

1. Abstract – The abstract would benefit from including quantitative outcomes and explicit statistical results to provide readers with a clearer understanding of the magnitude and significance of the findings.

Response: Bias-corrected effect sizes of significant frequency-specific mood impacts have been incorporated into the Abstract: "...In Study 1, 101 participants received 3, 6, 9, or 12 Hz BBs via a 250 Hz carrier. Only theta (6 Hz) BBs significantly enhanced calmness and focus ratings (Hedge’s g’s > .84; p < .001). Exposure to 9 Hz and 12 Hz BBs significantly enhanced calmness alone (g’s > .72; p’s < .001). In Study 2, 118 participants underwent 6 Hz BBs, 6 Hz BBs embedded in pink noise, pink noise alone, or silence. Both BB conditions significantly increased calmness and focus (g’s > .74; p’s < .001). BBs embedded in pink noise were identified to be subjectively preferable relative to isolated BBs, both of which were preferred to pink noise alone or silence (p’s < .001)..."

2. Highlights vs. Abstract – There is an apparent inconsistency between the Highlights and the Abstract. The Highlights state: “Mood gains elicited by theta BB stimulation were not replicated across pink noise or silent control conditions.” However, the Abstract suggests a beneficial role of pink noise: “BBs embedded in pink noise, pink noise alone, and silence. Both BB conditions significantly increased calmness and focus, with a subjective preference for BBs embedded in pink noise reflected by sentiment analysis.” The authors should clarify this discrepancy.

Response: The Highlight has been amended to clarify this discrepancy: "Mood gains elicited by theta BBs, whether presented in isolation or with a pink noise background, was not replicated across conditions presenting isolated pink noise or silence."

3. Introduction – The descriptions of Study 1 and Study 2 in the Introduction are unexpectedly detailed and would be more appropriate in the Methods section.

Response: The descriptions of both studies have been shortened in the Introduction by a third (pages 7-8).

4. Page 1 – The sentence “Study 2: One hundred and thirty undergraduate students … volunteered for the first study” appears to be a mistake. It should refer to the second study.

Response: The description has been corrected to: "...volunteered for the second study"

Figure 1 – The text within Figure 1 is too small and difficult to read. Increasing its legibility is recommended.

Response: Figure 1’s size and resolution has been increased to enhance legibility

6. Protocol description – The protocol is described multiple times throughout the manuscript, each time emphasizing different details. A more concise and unified description would improve clarity.

Response: The manuscript has been thoroughly revised to reduce redundancies in protocol descriptions. The revised manuscript contains a more concise justification of group conditions in the Introduction only (pages 7-8).

7. Acronym ROPE – ROPE appears several times in figure captions and tables before being defined (on pages 23 and 25). Acronyms should be defined at their first occurrence.

Response: ROPE is defined as the region of practical equivalence on first mention in Figure 3's caption only (page 19).

8. Acronym TOST – TOST is defined multiple times. It should be defined once at first use and referenced consistently thereafter.

Response: TOSTs is defined as two one-sided tests for equivalence on first mention on page 26 only.

9. Page 31, line 21 – The phrase “… in practice Third” - A period is missing at the end of the sentence.

Response: The period has been added.
